# Muscle ring finger-3 protects against diabetic cardiomyopathy induced by a high fat diet

**DOI:** 10.1186/s12902-015-0028-z

**Published:** 2015-07-28

**Authors:** Megan T. Quintana, Jun He, Jenyth Sullivan, Trisha Grevengoed, Jonathan Schisler, Yipin Han, Joseph A. Hill, Cecelia C. Yates, William E. Stansfield, Rudo F. Mapanga, M. Faadiel Essop, Michael J. Muehlbauer, Christopher B. Newgard, James R. Bain, Monte S. Willis

**Affiliations:** 1 Department of Surgery, University of North Carolina, Chapel Hill, NC USA; 2 Department of Pathology & Laboratory Medicine, University of North Carolina, Chapel Hill, NC USA; 3 General Hospital of Ningxia Medical University, Yinchuan, Ningxia People's Republic of China; 4 McAllister Heart Institute, University of North Carolina, Chapel Hill, NC USA; 5 Department of Biology, University of North Carolina, Chapel Hill, NC USA; 6 Department of Nutrition, University of North Carolina, Chapel Hill, NC USA; 7 Department of Pharmacology, University of North Carolina, Chapel Hill, NC USA; 8 North Carolina State University, Department of Engineering, Raleigh, NC USA; 9 Department of Internal Medicine (Cardiology), University of Texas Southwestern Medical Center, Dallas, TX USA; 10 Department of Health Promotions and Development, School of Nursing, University of Pittsburgh, Pittsburgh, PA USA; 11 Cardio-Metabolic Research Group (CMRG), Department of Physiological Sciences, Stellenbosch University, Stellenbosch, 7600 South Africa; 12 Sarah W. Stedman Nutrition and Metabolism Center, Duke Molecular Physiology Institute, Duke University Medical Center, Durham, NC USA; 13 Division of Endocrinology, Metabolism, and Nutrition, Department of Medicine, Duke University Medical Center, Durham, NC USA

**Keywords:** MuRF3, Diabetic cardiomyopathy, Post-translational modification, Multi-ubiquitin, PPAR, Ubiquitin ligase

## Abstract

**Background:**

The pathogenesis of diabetic cardiomyopathy (DCM) involves the enhanced activation of peroxisome proliferator activating receptor (PPAR) transcription factors, including the most prominent isoform in the heart, PPARα. In cancer cells and adipocytes, post-translational modification of PPARs have been identified, including ligand-dependent degradation of PPARs by specific ubiquitin ligases. However, the regulation of PPARs in cardiomyocytes and heart have not previously been identified. We recently identified that muscle ring finger-1 (MuRF1) and MuRF2 differentially inhibit PPAR activities by mono-ubiquitination, leading to the hypothesis that MuRF3 may regulate PPAR activity in vivo to regulate DCM.

**Methods:**

MuRF3−/− mice were challenged with 26 weeks 60 % high fat diet to induce insulin resistance and DCM. Conscious echocardiography, blood glucose, tissue triglyceride, glycogen levels, immunoblot analysis of intracellular signaling, heart and skeletal muscle morphometrics, and PPARα, PPARβ, and PPARγ1 activities were assayed.

**Results:**

MuRF3−/− mice exhibited a premature systolic heart failure by 6 weeks high fat diet (vs. 12 weeks in MuRF3+/+). MuRF3−/− mice weighed significantly less than sibling-matched wildtype mice after 26 weeks HFD. These differences may be largely due to resistance to fat accumulation, as MRI analysis revealed MuRF3−/− mice had significantly less fat mass, but not lean body mass. In vitro ubiquitination assays identified MuRF3 mono-ubiquitinated PPARα and PPARγ1, but not PPARβ.

**Conclusions:**

These findings suggest that MuRF3 helps stabilize cardiac PPARα and PPARγ1 in vivo to support resistance to the development of DCM.

MuRF3 also plays an unexpected role in regulating fat storage despite being found only in striated muscle.

**Electronic supplementary material:**

The online version of this article (doi:10.1186/s12902-015-0028-z) contains supplementary material, which is available to authorized users.

## Background

The MuRF3 (*Trim54*) ubiquitin ligase was the first muscle-specific RING-finger (MuRF) protein identified by its interaction with the serum response factor (SRF) transcription factor [1]. The three MuRF family members: MuRF1 (*Trim63*), MuRF2 (*Trim55*), and MuRF3 encode highly homologous proteins that both homo- and hetero-dimerize via their coiled-coil regions [2]. During muscle atrophy, MuRF1 and MuRF2 translocate to the nucleus in cardiomyocytes [3, 4] and act to inhibit gene expression via their regulation of transcription factors [5, 6]. MuRF1 and MuRF3 have been localized to the Z-disk [1, 2]. Both MuRF2 and MuRF3 co-localize with stable glutamylated microtubules during muscle assembly in vitro [1, 4] and in the early stages of cardiomyocyte sarcomere assembly in vivo [7, 8]. The association of MuRF proteins with microtubules suggests their potential role in regulating microtubule stability.

With expression limited to striated muscle, MuRF3 is a required protein for skeletal myoblast differentiation and support of the microtubule network [1]. Experimentally reducing MuRF3 expression during myocyte development results in a severe disruption of sarcomeric Z and M-band formation, likely due to these effects on tubulin dynamics [7]. MuRF3 expression increases postnatally and is found in both type I and type II muscle fibers [8]. The structural importance of MuRF3 has been demonstrated in MuRF3−/− mice, where a predisposition to cardiac rupture after MI has been reported [9]. Its role in the turnover of myosin heavy chain has been previously described. MuRF3 ubiquitinates myosin heavy chain, targeting it for degradation, indicating its role in myosin protein quality control [10]. The closely related MuRF1 family member similarly regulates myosin protein quality control; mice lacking both MuRF3 and MuRF1 exhibit the development of classic skeletal muscle myopathy, characterized by an accumulation of myosin [10]. Both diabetes and treatment with the chemotherapy doxorubicin increases cardiac MuRF3 expression (http://www.ncbi.nlm.nih.gov/geoprofiles/50107288) [11].

Initial studies in our laboratory identified that MuRF1 specifically regulates PPARα, but not PPARβ, or PPARγ activity both in vitro and in vivo [12, 13]. In the present study, we hypothesized that MuRF3−/− hearts would similarly regulate PPAR isoforms given the overlapping specificities of MuRF family proteins for specific substrates (myosin) and overlapping MuRF1−/−, MuRF2−/−, and MuRF3−/− altered cardiac metabolomics profiles recently identified by our laboratory [14]. With preliminary evidence that MuRF3−/− mice demonstrated increased levels of PPAR activity, we challenged them with a high fat diet model of diabetic cardiomyopathy. Since free fatty acids from the diet are the primary ligands for PPARs and one of the mechanisms driving diabetes induced cardiomyopathy, we hypothesized that MuRF3−/− mice would exhibit an enhanced cardiomyopathy and cardiac hypertrophy given MuRF3’s role in regulating PPARs and potentially SRF in vivo.

## Methods

### Animals and high fat diet-induced diabetic cardiomyopathy model

All experiments described used age-matched mice or littermates, male and female. All experiments were approved by the Institutional Animal Care and Use Committee (IACUC) review boards at the University of North Carolina and were performed in accordance with federal guidelines. MuRF3−/− mice, recently described and characterized [14], with strain-matched wildtype mice ~10 weeks of age were fed a high fat diet (60 % fat, 20 % protein, and 20 % carbohydrates) for 26 weeks as previously described [15]. Baseline body weight, blood glucose, serum insulin, serum triglyceride, and total cholesterol levels along with cardiac function were obtained prior to starting the diet. Mice receiving a high fat diet had body weight, blood glucose, and serum insulin levels measured every two weeks and echocardiography was performed every three weeks. An MRI was performed at baseline, 6, 12, and 22 weeks to detect body composition changes. After 26 weeks, mice were anesthetized with isoflurane, euthanized with cervical spine dislocation, and heart, liver, gastrocnemius, soleus, and tibialis anterior muscles were collected in cryovials, flash frozen, and stored at -80C.

### Mouse echocardiography

Conscious cardiac transthoracic echocardiography was performed on mice at the indicated time points using a VisualSonics Vevo 2100 ultrasound biomicroscopy system (VisualSonics, Inc., Toronto, Ontario, Canada). Investigators were blinded to mouse genotype. Two-dimensional M-mode echocardiography was performed in the parasternal long-axis view at the level of the papillary muscle on loosely restrained mice. Anterior and posterior wall thickness was measured as distance from epicardial to endocardial leading edges. Left ventricular internal diameters were also measured. Left ventricular systolic function was assessed by ejection fraction (LV EF% = [(LV Vol; d-LV Vol; s/LV Vol; d) × 100] and fractional shortening (%FS = [(LVEDD – LVESD)/LVEDD] × 100). Measurements represent the average of three cardiac cycles from each mouse.

### Body composition measurement

Conscious low-resolution nuclear magnetic resonance imaging was used to measure body composition of each mouse at baseline, 6, 12, and 22 weeks using an EchoMRI 3-in-1 Body Composition Analyzer for Live Small Animals (Mice)(EchoMRI, LLC, Houston, TX) [16]. Body fat and lean body mass was then calculated as a proportion of total body weight collected just prior to analysis as previously described [17].

### Blood collection, serum separation, and methods for glucose, insulin, triglyceride, and total cholesterol measurements

After overnight fast, ~200 μl whole blood was collected by submandibular vein lancet bleed (glucose) or brachial sinus puncture (remaining assays). One μl whole blood was analyzed via glucometer (PrecisionXtra, Abbott Diabetes Care Inc., Alameda, CA, USA) and test strip (Abbott Diabetes Care Ltd., Witney, Oxfordshire, UK). Blood collected in serum separator tubes for the remaining tests was incubated on ice for 90 min, and centrifuged at 1600 × g (20 min at 4 °C). Insulin levels were measured using the Insulin Enzyme Immunoassay Kit (Cayman Chemical, Cat.#589501, Ann Arbor, MI 48108) according to the manufacturer’s instructions as previously described [18]. Serum triglyceride and cholesterol levels were measured using an automated chemical analyzer (Vitro 350, OrthoClinical Diagnostics Company, Rochester, NY).

### Fatty acid extraction and triglyceride assay

Fatty acid extraction and tissue triglyceride concentrations were determined on flash frozen heart tissue, liver tissue, and skeletal tissue as previously described [19]. Briefly, 25–50 mg of heart, liver and skeletal muscle was homogenized 15–30 s with a bladed homogenizer (Power Gen 125, Cat.#14-261, setting 6, Fisher Scientific, Inc., Pittsburgh, PA) in 10X (v/w) ice cold lysis buffer (20 mM Tris base, 1 % Triton-X100, 50 mM NaCl, 250 mM NaF, 5 mM Na4P2O7-10H2O, 1 tablet protease inhibitor (Roche Inc., Cat.#11836153)) and incubated at 4 °C for 1 h. Two hundred μl of homogenate was transferred to chloroform resistant tubes, mixed with 0.4 ml methanol and 0.8 ml chloroform, placed on the rocker at 4 °C for at least 30 min. Potassium chloride (0.24 ml 0.88 % KCl) was added, samples vortexed, and centrifuged at 1000 × g for 15 min at 4 °C. The bottom layer of CHCl_3_ was then transferred and this process was repeated with another 0.8 ml of chloroform and the combined CHCl_3_ layers were then dried under N_2_. One hundred μl of a tert-butanol:methanol:Triton X-100 solution (3:1:1, v/v/v) was added to each tube and samples were stored at −20 °C. Glycerol standard 2.5 mg/dl (Sigma, Inc., Cat.#G1394), free glycerol reagent (Sigma Aldrich, Inc., Cat.#F6428) and triglyceride reagent (Sigma Aldrich, Inc., Cat.#T2449) were used to measure triglyceride concentrations. Five μl of samples were added to a 96-well plate. Working reagent was added to the samples (4 volumes of free glycerol reagent: 1 volume of triglyceride reagent). This was left to incubate, rocking, at room temperature for 15 min. Then absorbance was measured per sample at 540 nm using the Clariostar High Performance Multimode Microplate Reader (BMG LABTECH, San Francisco, CA) and normalized to tissue weight.

### Tissue glycogen assay (Acid Hydrolysis Method)

Tissue glycogen was measured from heart, liver and skeletal muscle using a colorimetric tissue glycogen assay kit (Sigma, Inc., Cat.#G3293) as previously described [20]. Briefly, 15–25 mg of tissue was powdered in liquid nitrogen, collected in a pre-chilled 2 ml tube, 0.5 ml 1 N HCl added, then homogenized with a bladed homogenizer (Fisher Scientific, Power Gen 125, Cat.#14-261, setting 6, Pittsburgh, PA) under a hood. The resulting homogenate (100 μl) was quickly added to 100 μl 1 N NaOH and kept on ice until heated in HCl at 95 °C for 90 min, mixing every 30 min, cooled to RT and 0.4 ml 1 N NaOH was added to neutralize the sample. After the sample was centrifuged at 14,000 × g for 10 min at RT, the supernatant was used for glucose analysis using a hexokinase-dependent assay kit (Sigma, Inc., Cat.#G3293) according to the manufacturer’s instructions. Briefly, 10 μl (liver) or 20 μl (heart and gastrocnemius) of supernatant was put into a 96-well plate, mixed with 200 μl of reagent, incubated at room temperature for 15 min, and the absorbance was measured at 340 nm.

### RNA isolation and quantitative PCR analysis of PPAR-regulated gene expression

Total RNA was isolated using TRIzol reagent according to the manufacturer’s protocols (Life Technologies, Inc., Cat.#15596-026). About 25 mg of cardiac ventricular tissue was put into TRIzol reagent and homogenized on ice (Fisher Scientific, Power Gen 125, setting 5). Total mRNA expression was determined using a two-step reaction. cDNA was made from total RNA using the iScript™ Reverse Transcription Supermix for RT-qPCR kit (Cat.#170-8841, BIO-RAD), with a total volume of 20 μl per reaction. The complete reaction mix was incubated in an Eppendorf Cycler (Hamburg, Germany) using the following protocol: priming 5 min at 25C, reverse transcription 30 min at 42C, RT inactivation 5 min at 85C. PCR products were amplified on a Roche Lightcycler 480IIsystem using cDNA, Taqman Probes (Applied Biosciences™), and Lightcycler 480 Probe Master Mix 2X (Cat.#04 707 494 001). The TaqMan probes used in this study were Mm00430615_m1 (ACC1), Mm00443579_m1 (ACOX1), Mm00475794_m1 (ADRP), Mm00599660_m1 (LCAD), Mm00431611_m1 (MCAD), Mm00440939_m1 (PPARα), Mm01305434_m1 (PPARβ), Mm00443325_m1 (PDK4), Mm00487200_m1 (CPT1b), Mm00441480_m1 (Glut1, Slc2a1), Mm01245502_m1 (Glut4, Slc2a4), Mm01309576_m1 (PFK), Mm00432403_m1 (CD36, FAT), Mm01185221_m1 (MuRF1, Trim63), and Mm01292963_g1 (MuRF2, Trim55), Mm00491308_m1 (MuRF3, Trim54), Hs99999901_s1(18S), Mm00440359_m1(α-MHC, Myh6), Mm00600555_m1(β-MHC, Myh7), Mm01255747_g1(ANP), Mm00435304_g1(BNP), Mm00808218_g1(SK α-actin) (Applied Biosystems, Inc., Foster City, CA). Assay of PPARγ1 was performed using the Roche Universal Probe technology, including forward primer (gggctgaggagaagtcacac) and reverse primer (gggctgaggagaagtcacac) in conjunction with UPL probe #92 (Roche, Inc., Cat.#04692098001). Samples were run in triplicate and relative mRNA expression was determined using 18S as an internal endogenous control. RNase-free water, 2X Master Mix, Taqman Probe or Roche UPL primer and probe, cDNA were used for each reaction.

### Western blot

Western analysis of ventricular tissue was performed on lysates created from ~25 mg tissue. Tissue lysates in Lysis Buffer (Cell Signaling, Cat.#9803S) with XM β-glycerol phosphate (Sigma, Cat.#G6251), protease inhibitor (Roche, Cat.#11 836 153 001), and phosphatase inhibitor cocktail (Roche Cat.#04 906 837 001) were manually homogenized on ice (Fisher Scientific, Power Gen 125, setting 5) for 15–20 s. Alternatively, tissue was placed in 8 M Urea Lysis Buffer (8 M Urea Sigma, Cat. #U0631, 5 M NaH_2_PO_4_ Sigma, Cat. #S3139, 1 M Tris-Cl pH 8.0) at a ratio of 15 μl lysis buffer/mg of tissue and was homogenized at 4C (TissueLyser LT, Qiagen, Cat. #85600) for 2 min. Homogenates were incubated on ice for 30 min, centrifuged at 16,000 × g (4C) for 15 min and the supernatant stored at -80C. Protein concentration was determined using the Bio-Rad DC Protein Assay Reagent Package (Bio-Rad Laboratories, Inc., Hercules, CA, Cat.#500-0116). Proteins (30–50 μg/lane) were resolved on NuPAGE Bis-Tris or Tris-Acetate 10 well gels. Mouse anti-NFκB p65, rabbit anti-phospho-NFκB p65 (Ser536), rabbit anti-phospho-NFκB p65 (Ser468) were used to measure NFκB signaling (Cell Signaling Technologies, Cat.#4767, each 1:500). IRS-1 signaling was detected using rabbit anti-phospho-IRS-1 (Ser1101) and rabbit anti-IRS-1 (Cell Signaling Technologies, Inc. Cat.#2385 and #2383, each 1:500). cJun signaling was detected by rabbit anti-p-cJun (Ser73), rabbit anti-p-cJun (Thr91) or rabbit anti-cJun 60A8 (Cell Signaling Technologies, Cat.#9164, #2303, #9165, each 1:500). Primary antibodies were diluted in 4 % BSA/TBS-T and incubated at 4 °C overnight. HRP-labeled secondary antibodies against mouse (Sigma #A9917, 1:10,000) and rabbit (Sigma #A9169, 1:5,000) were used to detected the primary antibodies diluted in 1X TBS-T and incubated 1 h at room temperature. Mouse anti-β-actin (Sigma, Inc., Cat.#A2228, 1:4000) was used as a loading control throughout. Secondary antibody HRP was detected using ECL Select (GE Healthcare, Cat.#RPN2235) and imaged using the MultiDoc-it Imaging System (UVP, LLC Ultra-violet Products, Ltd., Upland, CA).

### Total *O*-GlcNAc expression

Total *O*-GlcNAc expression was determined by SDS-PAGE as previously described [21], using anti-*O*-GlcNAc (RL-2, Santa Cruz Biotechnology, Santa Cruz CA) on PVDF blocked with 1 % bovine serum albumin dissolved in TBS-T solution for 20 min, followed by an overnight incubation with *O*-GlcNAc antibody (1:1000) at 4 °C. Secondary antibody (goat-anti-mouse IgG-HRP, Santa Cruz Biotechnologies, Santa Cruz CA; 1:4000) incubated for 1 h at RT, washed with TBS-T, then visualized with enhanced chemiluminescence (ECL) on the ChemiDoc™ XRS+ system with Image Lab™ Software v2.0 (Bio-Rad Laboratories, Hercules CA). Total *O*-GlcNAcylation (per lane) was quantified by the adjusted percentage volume - intensity units of pixels of band × mm^2^ - after background subtraction using Quantity One Software v4.6.9 (Bio-Rad Laboratories, Hercules CA), and normalized to β-actin (Abcam, Cambridge MA).

### In vitro ubiquitination assay

Human recombinant GST-E1 (50 nM, Boston, Biochem, Cambridge, MA, Cat.#E-306), human recombinant UbcH5c/UBE2D3 (2.5 μM, Boston Biochem, Inc., Cambridge, MA, Cat.#E2-627), human recombinant ubiquitin (250 μM, Boston Biochem, Inc., Cat.#U-100H), human MuRF3 recombinant protein (1 mg, LifeSensors, Cat.#UB306, Malvern, PA), human PPAR-α, −β, and -γ recombinant protein (500 ng, Sigma-Aldrich, Inc., St. Louis, MO, Cat.#SRP2043, Cat.#SRP2044, and Cat.#SRP2045, respectively) were added to reaction buffer (50 mM HEPES, pH 7.5) containing 5 mM MgATP solution (Boston Biochem, Inc., Cat.#B-20) and 0.6 mM DTT, then incubated at 37 °C for 1 h. The reaction was stopped by adding SDS-PAGE sample buffer and heating, then resolved on a 4-12 % Bis-Tris gel with MOPS running buffer (Invitrogen Corp.) and transferred to PVDF membranes for immunoblotting with goat polyclonal anti-MuRF3 antibody (Cat.#sc-50252, Santa Cruz Biotechnology), rabbit polyclonal anti-PPARα antibody (Cat.#Ab24509, Abcam), rabbit polyclonal anti-PPARβ antibody (Cat.#AB10094, Millipore), or rabbit polyclonal anti-PPARγ antibody (Cat.#2443, Cell Signaling Technology).

### Histology and cross-sectional area analysis

Hearts were perfused with 4 % paraformaldehyde, fixed for 24 h, moved to 70 % ethanol, then process and embedded in paraffin to be cut in 5 mm sections. Slides were stained with H&E and Masson’s Trichrome (MT) using standard procedures. Imaging of H&E and MT-stained sections was obtained using Aperio Scanscope and Aperio Imagescope software (version 10.0.36.1805, Aperio Technologies, Inc., Vista, CA). MT-stained images were taken using Aperio Imagescope (TIFF) and analyzed using NIH ImageJ using Aperio exported image ruler. Cardiomyocyte cross-sectional area was measured using Image J software. A minimum of 25 random fields of the left ventricle at × 200 magnification were imaged from at least three different sections from three biological replicates per experimental group.

### Cross-sectional area analysis and fibrosis (%) determination

Sections of the MT-stained tissue were analyzed in four cross-sectional defined areas for arrangement and collagen content histologically and quantitatively by using Meta-Morph analysis (Molecular Devices). Controls served to set the threshold against which the MuRF3−/− mouse sections were measured. Immunostaining for vimentin was performed as described previously [22–24]. Briefly, cardiac sections were stained with antibodies against Vimentin (1:100, Cat. #SC-6260, Santa Cruz, Dallas, TX) or an irrelevant isotype mouse IgG (as a negative control) at 4 °C overnight. Section were then treated with Alexa Fluor 488-conjugated secondary antibodies and counterstained with 4,6-diamidino-2-phenylindole (DAPI)(Vector Laboratories, Burlingame, CA). Total positive vimentin stain cells were identified and counted in each of the four cross-sectional defined areas using Meta-Morph software (Molecular Devices). Images were taken using EVOS XL Core cell imaging system (Life technologies).

### Non-targeted metabolomics determination by GC-MS Instrumentation

Cardiac tissue was flash frozen with liquid nitrogen cooled in a biopress, a fraction weighted (~25-30 mg weight), finely ground and added to fresh 50 % acetyl-nitrile, 50 % water, 0.3 % formic acid at a standard concentration of 25 mg/475 mcl buffer then fully homogenized on ice for 10–25 s and placed on dry ice/stored at −80 °C. Samples were “crash” deprotonized by methanol precipitation and spiked with D27-deuterated myristic acid (D27-C14:0) as an internal standard for retention-time locking and dried. The trimethylsilyl (TMS)-D27-C14:0 standard retention time was set at *16.727 min. Reactive carbonyls were stabilized at 50 °C with methoxyamine hydrochloride in dry pyridine. Metabolites were made volatile with TMS groups using N-methyl-N-(trimethylsilyl) trifluoroacetamide or MSTFA with catalytic trimethylchlorosilane at 50 °C. GC/MS methods generally follow those of Roessner *et al.* (2000) [25], Fiehn *et al.* (2008) [26], and Kind *et al.* (2009) [27], and used a 6890 N GC connected to a 5975 inert single quadrupole MS (Agilent Technologies, Santa Clara, CA). The two wall-coated, open-tubular (WCOT) GC columns connected in series were both from J&W/Agilent (part 122–5512), DB5-MS, 15 m in length, 0.25 mm in diameter, with an 0.25-lm luminal film. Positive ions generated with conventional electron-ionization (EI) at 70 eV were scanned broadly from 600 to 50 m/z in the detector throughout the 45 min cycle time. Data were acquired and analyzed as previously described [14, 28].

### Statistical analysis

Sigma Plot 11.0 and Prism were used to plot and statistically analyze data. Depending upon the experimental design, several statistical tests were applied to the studies. Student’s *t*-test or One Way ANOVA followed by Holm-Sidak pairwise post-hoc analysis was performed, indicated in the figure legends. Significance was determined as a p < 0.05. Values are expressed as mean ± SE. Statistical analysis on metabolomics data was performed as previously described [14, 28]. Metaboanalyst (v2.0) run on the statistical package R (v2.14.0) used metabolite peaks areas (as representative of concentration) [29, 30]. These data were first analyzed by an unsupervised principal component analysis (PCA), which identified the presence of the MuRF3 −/− after 26 weeks high fat diet as the principal source of variance. To sharpen the separation between our two groups, data were next analyzed using a partial least squares discriminant analysis (PLS-DA) to further determine which metabolites were responsible for separating these two groups. The specific metabolites contributing most significantly to the differences identified by PLS-DA between MuRF3−/− and wildtype control group hearts were determined using the variable importance in projection (VIP) analysis in the metaboanalyst environment. The metabolites that best differentiated the groups were then individually tested using the Student’s *t*-test (Microsoft Excel 2011, Seattle, WA). The VIP and *t*-test significant metabolites were matched to metabolomics pathways using the Pathway Analysis feature in Metaboanalyst 2.0. Heat maps of the metabolite data (individual and grouped) were generated using the GENE E software (http://www.broadinstitute.org/cancer/software/GENE-E/index.html).

## Results

**Fig. 1:**
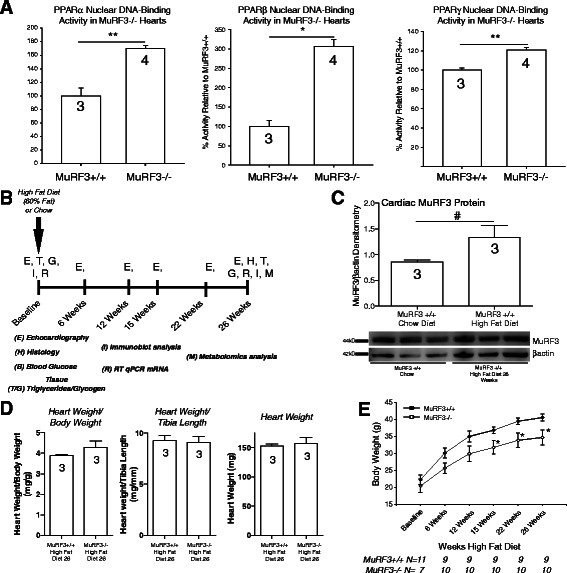
Role of MuRF3 in regulating PPAR isoform activity and its role in high fat diet cardiac hypertrophy in vivo. Isolation of cardiac nuclei from MuRF3−/− and sibling wildtype mouse hearts revealed increases in **a**. PPARα, PPARβ/δ, and PPARγ activity using PPRE-DNA as bait and ELISA detection of PPARα protein (N = 3-4/group as indicated in bars). **b**. Experimental design of high fat diet (60 %)-induced cardiomyopathy. **c**. High fat diet induces cardiac MuRF3 levels after 26 weeks HFD (N = 3/group). **d**. MuRF3−/− heart weights 26 weeks high fat diet normalized to body weight and tibia length (N = 3/group). **e**. MuRF3−/− body weight at baseline and after high fat diet for 26 weeks (N indicated below graph). Values expressed as Mean ± SE. Statistical analysis was performed using a Student’s *t*-test comparing MuRF3−/− and MuRF3+/+ groups. *p ≤ 0.001, **p < 0.01. #p < 0.05

Initial studies in our laboratory identified that MuRF1 regulated PPARα, but not PPARβ, or PPARγ using PPAR-response element (PPRE)-DNA binding assays [12, 13]. This led us to characterize PPAR isoform activities in our MuRF3−/− mouse model. We hypothesized that MuRF3−/− hearts would similarly regulate PPARα given the overlapping specificities of MuRF family proteins. Cardiac nuclei were isolated from MuRF3−/− hearts and assayed for PPRE-DNA binding activity by ELISA, followed by specific recognition of PPARα, PPARβ, or PPARγ (Fig. 1a). To our surprise, all three PPAR isoforms were significantly elevated in MuRF3−/− hearts compared to sibling wildtype controls, with +60 % increase in PPARα, 300 % increase in PPARβ, and +20 % increase in PPARγ in unchallenged mice on a chow diet (Fig. 1a).

Given the critical role of cardiac PPARα, PPARβ, and PPARγ1 in diabetic cardiomyopathy [31], we next challenged MuRF3−/− mice to a high fat diet (60 % fat, 20 % protein, and 20 % carbohydrates) for 26 weeks (Fig. 1b) as previously described [15]. In the context of diabetic cardiomyopathy, we found that wildtype hearts exhibited a 50 % increase in MuRF3 expression 26 weeks post-high fat diet (Fig. 1c), a point in which insulin resistance and hypertrophy were identified (described below). MuRF3−/− and wildtype hearts did not significantly differ in heart weight after 26 weeks high fat diet (Fig. 1d), but MuRF3−/− mice had significant reductions in body weight compared to strain-matched MuRF3+/+ mice (Fig. 1e).

**Fig. 2:**
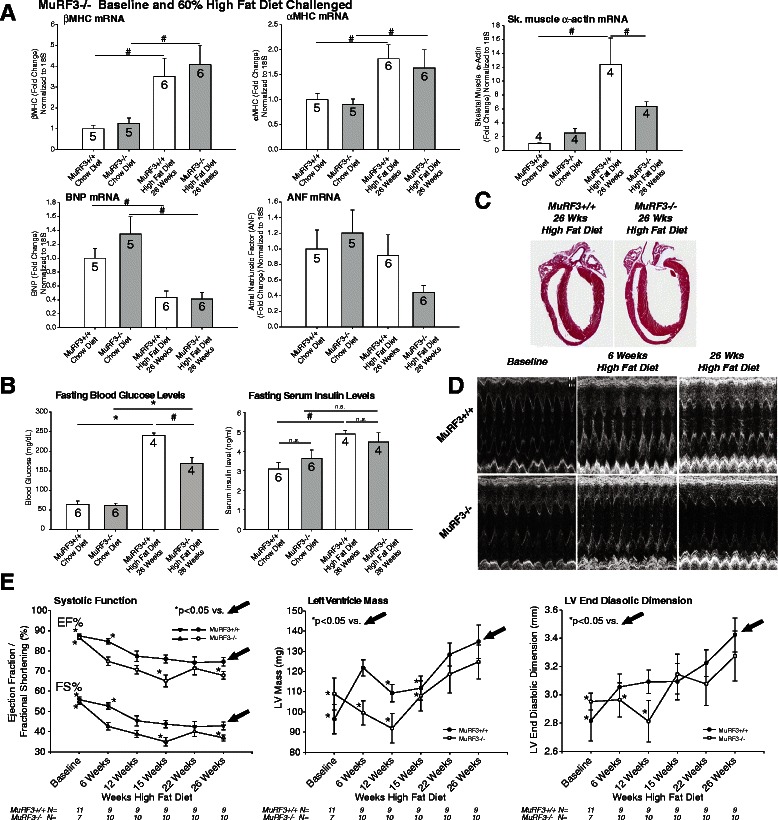
MuRF3−/− experience significant deficits in cardiac function after challenge with high fat diet. **a** qPCR analysis of heart failure associated fetal gene expression in MuRF3−/− mice at baseline and after 26 weeks high fat diet challenge. **b** Fasting blood glucose and fasting serum insulin levels. **c** Histological analysis of MuRF3−/− and wildtype hearts post-high fat diet challenge. **d**/**e** MuRF3−/− mice exhibit an accelerated heart failure by 6 weeks after the initiation of the high fat diet. Values expressed as Mean ± SE. N indicated in bars (**a** & **b**) or below graph (**e**). The significance of observed differences in grouped mean values was determined using a One Way ANOVA followed by Holm-Sidak pairwise post hoc analysis. *p < 0.001, #p < 0.05

To further delineate the pathological cardiac hypertrophy in MuRF3−/− mice, RT-qPCR analysis of fetal gene expression was performed. As commonly found in the literature, αMHC was increased predictably along with βMHC after 26 weeks of high fat diet, but did not differ between MuRF3−/− and wildtype controls (Fig. 2a). Significant reduction in brain natriuretic peptide (BNP) was identified as reported in diabetic models of cardiomyopathy (Fig. 2a) [32]. Mechanisms that have been implicated in driving diabetic cardiomyopathy include hyperinsulinemia, which acts to stimulate PI3Ka/Akt-1 pathways by which it mediates glucose uptake [33]. Akt-1 also activates mTOR, downstream p70, and S6kinase-1 to increase protein synthesis [33]. Therefore, we next investigated blood glucose and serum insulin levels in fasting mice in MuRF3−/− and wildtype controls (Fig. 2b). As expected, 26 weeks of high fat diet resulted in increased fasting glucose and insulin levels (wildtype mice only), consistent with insulin resistance. MuRF3−/− mice, however, showed significantly lower blood glucose at 26 weeks high fat diet. Both MuRF3−/− and wildtype mice exhibited significant increases after 26 weeks high fat diet. Interestingly, at that time point, the MuRF3−/− mice demonstrated significantly reduced glucose levels compared to wildtype mice. This indicates that they are more insulin sensitive.

As previously reported, MuRF3−/− hearts at baseline did not differ from sibling wildtype controls [9, 14]. Despite the lack of histological findings (Fig. 2c, Additional file 1: Figure S1), MuRF3−/− hearts experienced significant decreases in cardiac function by 6 weeks high fat diet (Fig. 2d, 2e). Increases in cardiac LV mass were observed after high fat diet, but when normalized for body weight, MuRF3−/− hearts were not significantly increased after 26 weeks HFD (Fig. 2e, middle panel). Both MuRF3−/− and wildtype hearts exhibited a progressive LV dilation consistent with heart failure, evidenced by increases in LV end diastolic dimension (Fig. 2e, right panel). With the dynamic of heart failure and increasing cardiac mass, increases in anterior and posterior wall thicknesses essentially did not increase over time (Table 1), consistent with the pathogenesis of diabetic cardiomyopathy and characteristic cardiac hypertrophy [32, 34, 35].

**Table 1:** High-resolution transthoracic echocardiography performed on conscious MuRF3−/− and age-matched wild type mice at baseline, 6 weeks, 12 weeks, 15 weeks, 22 weeks, and 26 weeks high fat diet. Data represent means ± SEM. A One Way ANOVA was performed (¶p < 0.05), followed by Holm-Sidak Multiple Comparison vs. Control Group (MuRF3+/+ 26 Weeks High Fat Diet)

												
	MuRF3	MuRF3	MuRF3	MuRF3	MuRF3	MuRF3	MuRF3	MuRF3	MuRF3	MuRF3	MuRF3	MuRF3
	+/+	-/-	+/+	-/-	+/+	-/-	+/+	-/-	+/+	-/-	+/+	-/-
	Baseline	Baseline	6 Wks High Fat Diet	6 Wks High Fat Diet	12 Wks High Fat Diet	12 Wks High Fat Diet	15 Weeks High Fat Diet	15 Weeks High Fat Diet	22 Wks High Fat Diet	22 Wks High Fat Diet	26 Weeks High Fat Diet	26 Weeks High Fat Diet
	N=11	N=7	N=9	N=10	N=9	N=10	N=9	N=10	N=9	N=10	N=9	N=10
	(1)	(2)	(3)	(4)	(5)	(6)	(7)	(8)	(9)	(10)	(11)	(12)
AWTD (mm)	1.03±0.02	1.07±0.03	1.14±0.04	0.98±0.04	1.03±0.04	1.01±0.03	1.04±0.04	1.02±0.04	1.09±0.04	1.09±0.02	1.08±0.03	1.06±0.04
AWTS (mm)	1.65±0.04	1.68±0.05	1.78±0.05	1.59±0.03	1.62±0.09	1.60±0.05	1.71±0.06	1.54±0.07	1.66±0.04	1.68±0.04	1.71±0.04	1.60±0.05
LVEDS (mm)¶	1.25±0.08*	1.33±0.03*	1.45±0.07*	1.70±0.12	1.70±0.12	1.73±0.12	1.75±0.12	2.05±0.12	1.86±0.10	1.85±0.13	1.95±0.06	2.08±0.14
PWTD (mm)	0.99±0.03	1.01±0.06	1.05±0.04	1.00±0.03	0.99±0.04	0.94±0.03	1.00±0.03	0.93±0.04	1.06±0.03	1.03±0.05	1.01±0.04	1.01±0.04
PWTS (mm)¶	1.65±0.07	1.64±0.06	1.63±0.05	1.43±0.07	1.52±0.09	1.37±0.07	1.51±0.09	1.31±0.04*	1.49±0.07	1.46±0.08	1.50±0.05	1.37±0.07
LV Vol;d (μl)	31.3±3.9	33.8±1.7	37.0±2.7	34.8±3.5	38.0±2.4	31.1±4.0	38.6±3.8	40.5±4.3	42.2±2.9	38.6±4.8	49.0±4.1	45.1±6.1
LV Vol;s (μl)¶	4.1±0.7*	4.5±0.3*	5.8±0.7*	8.8±1.3	9.0±1.5	9.5±1.8	9.8±1.7	14.2±2.0	11.0±1.4	11.3±2.2	12.0±0.9	15.1±2.6
LV Mass/BW (mg/g)	4.33±0.21*	5.42±0.29*	4.11±0.21*	3.89±0.11*	3.16±0.15	3.16±0.25	3.04±0.16	3.40±0.10	3.18±0.10	3.65±0.16	3.35±0.21	3.78±0.17
HR (bpm)	629±15	619±23	676±6	664±13	681±8	674±9	694±8	697±13	656±19	665±7	662±15	659±9

HR, heart rate; ExLVD, external left ventricular diameter; bpm, heart beats per minute; AWTD, anterior wall thickness in cxxdiastole; AWTS, anterior wall thickness in systole; PWTD, posterior wall thickness in diastole; PWTS, posterior wall thickness in systole; LVEDD, left ventricular end-diastolic dimension; LVESD, left ventricular end-systolic dimension; FS, fractional shortening, calculated as (LVEDD-LVESD)/LVEDD x 100; EF%, ejection fraction calculated as (end Simpson’s diastolic volume – end Simpson’s systolic volume)/end Simpson’s diastolic volume * 100

*p < 0.05 vs. MuRF3+/+ 26 Wks High Fat Diet ()

**Fig. 3:**
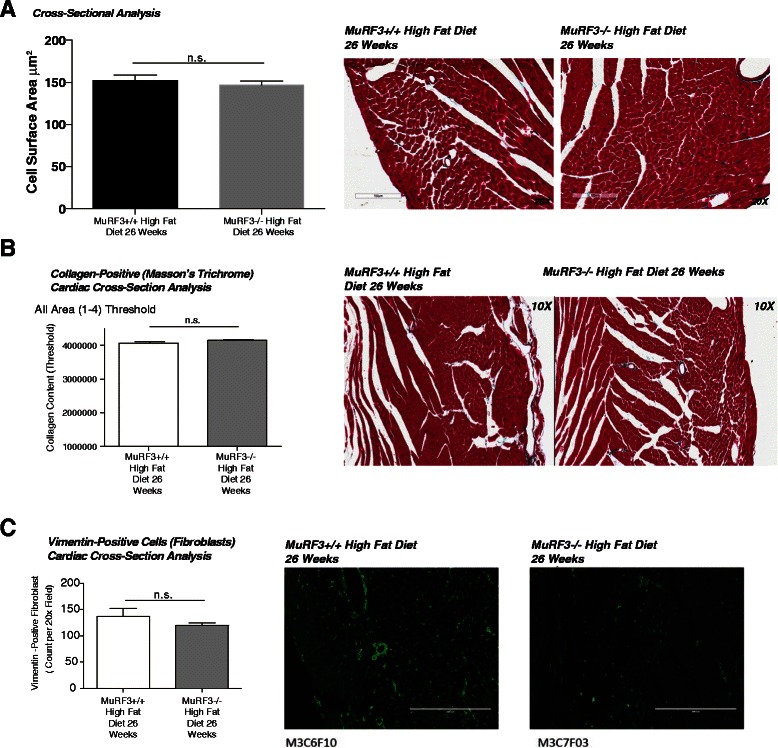
Histological analysis of cardiac fibrosis. **a** Cross-sectional analysis of myocytes reveal no differences between MuRF3−/− and wildtype hearts (Masson’s Trichrome). **b** Objective blinded computer analysis of fibrosis of MuRF3−/− and wildtype hearts after 26 weeks high fat diet reveals no significant differences. **c** Confocal immunofluorescence analysis of vimentin (fibroblasts) in cardiac cross-sections from MuRF3−/− mice after 26 weeks HFD. Values expressed as Mean ± SE. N = 3 WT and N = 3 MuRF3−/−. A Student’s *t*-test was performed comparing MuRF3−/− vs. MuRF3+/+ groups. #p < 0.05

To further characterize the cardiac hypertrophy in the MuRF3−/− hearts after 26 weeks high fat diet, we next did an analysis of cardiomyocyte cross-sectional areas in perfused fixed histological sections. No differences were identified when measurements were taken from multiple animals across multiple levels (Fig. 3a). The hyperglycemia of diabetes can contribute to cardiac injury by multiple mechanisms, including direct and indirect effects on cardiomyocytes and cardiac fibroblasts. One hallmark of human diabetic cardiomyopathy is fibrosis [36, 37], so we next investigated fibrosis by analyzing Masson’s Trichrome stained cross-section using an objective logarithm to analyze blue collagen content (Fig. 3b). Only minimal amount of fibrosis was detected, with no differences between MuRF3−/− and wildtype controls identified. Analysis of cardiac fibroblast numbers by immunofluorescent staining of vimentin positive cells similarly found no differences in the number of fibroblasts present in MuRF3−/− and wildtype controls (Fig. 3c). In a broader context, these findings illustrate that increased susceptibility of MuRF3−/− hearts to cardiac hypertrophy seen after 26 weeks high fat diet is not due to changes in fibrosis or underlying differences in cardiac fibroblast numbers.

**Fig. 4:**
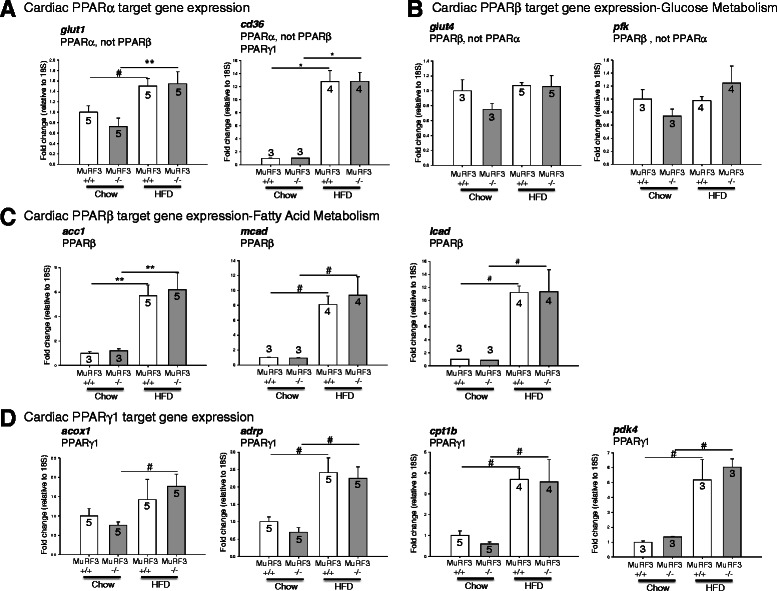
High fat diet-induced increases in PPAR-regulated gene (mRNA) levels in MuRF3−/− hearts. RT-qPCR analysis of cardiac **a**. Cardiac PPARα target gene expression, **b**. PPARβ-regulated mRNA target genes involved in glucose metabolism, **c**. PPARβ-regulated mRNA target genes involved in fatty acid metabolism. **d** PPARγ1-regulated mRNA target genes. Values expressed as Mean ± SE. The significance of observed differences in grouped mean values was determined using a One Way ANOVA followed by Holm-Sidak pairwise post hoc analysis. N per group indicated above graph. *p ≤ 0.001, **p < 0.01, #p < 0.05

PPAR isoform-specific gene expression has been described in PPARα, PPARβ, and PPARγ1-specific transgenic mouse models [38–40]. While gene overlap exists, multiple genes are reported to be increased in PPARα, but not PPARβ (Fig. 4a). Similarly, multiple genes have been reported increased in PPARβ but not PPARα transgenic hearts (Fig. 4b) [38–40]. PPARβ and PPARγ1 genes similarly have been described (Fig. 4c, d, respectively [38–40]). In response to high fat diet, both MuRF3−/− and wildtype control hearts had increased expression of the PPARα target genes *glut1* and *cd36* mRNA by RT-qPCR analysis (Fig. 4a). Increases in PPARβ fatty acid metabolism genes (Fig. 4c), but not PPARβ glucose metabolic genes (Fig. 4b) were identified. Both MuRF3−/− and wildtype hearts showed increases in PPARγ1 target genes 26 weeks after high fat diet challenge (Fig. 4d). Notably, MuRF3−/− expression levels did not significantly differ from sibling wildtype control hearts in any of the genes investigated (Fig. 4). Together, these studies illustrate that the increases in cardiac mass present in the MuRF3−/− mice after 26 weeks high fat diet were not due to differences in PPAR-driven gene expression between the two groups.

**Fig. 5:**
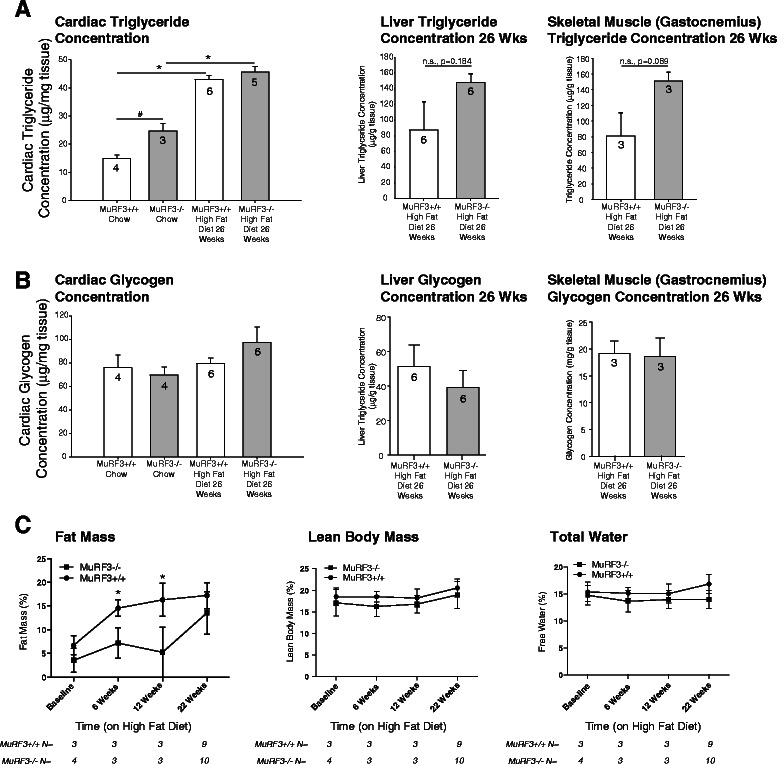
Analysis of tissue triglyceride, glycogen, and fat mass in MuRF3−/− mice after high fat diet challenge. **a** Triglyceride analysis of cardiac left ventricle (LV), liver, and skeletal muscle (gastrocnemius). **b** Glycogen analysis of cardiac LV, liver, and skeletal muscle (gastrocnemius). **c** Magnetic resonance imaging (MRI) analysis of fat mass, lean body mass, and free water at baseline, 6, 12, and 22 weeks HFD. Values expressed as Mean ± SE. A one-way ANOVA was performed to determine significance of cardiac LV triglyceride and glycogen concentrations, followed by a Holm-Sidak pairwise comparison to determine significance between groups. A Student’s *t*-test was performed comparing MuRF3−/− vs. MuRF3+/+ groups. Numbers above bars represent number of animals (N) included in each experiment (N = MuRF3+/+, MuRF3−/− in **c**). n.s. = not significant. *p < 0.001, **p < 0.01, #p < 0.05

The toxicity of diabetes to the heart has been attributed to increases in cardiac triglyceride content and the mishandling of cardiac glycogen [41–45]. Since MuRF3 has been reported in skeletal muscle as well as cardiomyocytes [10], we next did an analysis of cardiac triglyceride and gastrocnemius muscle as well as liver as a control. Consistent with the free fatty acid upregulation of PPAR-regulated fatty acid oxidation and storage seen in our initial experiments, significant increases in cardiac triglyceride were identified 26 weeks after high fat diet challenge (Fig. 5a). With comparable significant increases in serum cholesterol and triglycerides (Additional file 1: Figure S1B) both MuRF3−/− and wildtype hearts exhibited increased accumulation of cardiac triglyceride to the same extent (Fig. 5a, left panel). Differences in liver and skeletal muscle triglyceride were not identified (Fig. 5a). No increases in glycogen stores were seen after high fat diet in the heart, liver, or representative skeletal muscle (Fig. 5b). MRI analysis of fat mass, lean body mass, and total water of MuRF3−/− and wildtype mice were performed at baseline and after 6, 12, and 22 weeks of high fat diet (Fig. 5c). Consistent with these findings, gastrocnemius, soleus, and tibialis anterior weights did not differ (Additional file 1: Figure S1C). Interestingly, MuRF3−/− mice were resistant to increases in fat mass resulting from the high fat diet intake at 6 and 12 weeks, despite recovering these differences by 22 weeks (Fig. 5c). These studies illustrate that the MuRF3−/− cardiac hypertrophy and heart failure in diabetic cardiomyopathy cannot be explained by differential storage of cardiac triglyceride or glycogen and that differences in fat mass were relatively transient.

**Fig. 6:**
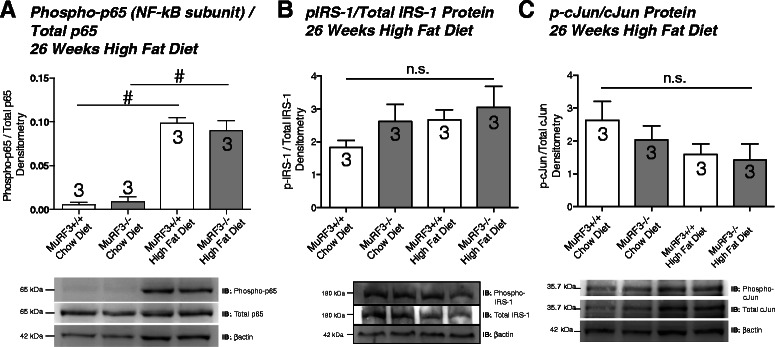
Western analysis of MuRF3−/− cardiac NF-κB, IRS-1, and cJun signaling. Immunoblot analysis of **a**. NF-κB, **b**. IRS-1, and **c**. cJun reveal no differences in MuRF3−/− and sibling wildtype mice. Values expressed as Mean ± SE. Statistical analysis was performed using a Student’s *t*-test. N = 3/group. n.s. = not significant. #p < 0.05

Recent studies have implicated the generation of ROS and activation of NF-κB signaling in the pathogenesis of diabetic cardiomyopathy [34, 46, 47]. To determine if inflammatory signaling or differences in insulin resistance were present to a different degree in MuRF3−/− hearts, we quantified cardiac phospho-p65 indicative of activated NF-κB, pIRS-1, indicative of insulin signaling, and phospho-cJun as a measure of downstream JNK signaling resulting from oxidative stress [48]. While high fat diet clearly increased NF-κB activation (phospho-p65/total p65 protein levels) in both MuRF3+/+ and MuRF3−/− mice compared to chow diet (Fig. 6a, right two groups (chow) increased vs. left two groups (HFD challenged), MuRF3−/− cardiac phospho-p65 levels increased to the same level as wildtype hearts (Fig. 6a, far right group vs. MuRF3 +/+ high fat diet). No differences in phospho-IRS-1 or p-cJun were identified after 26 weeks high fat diet challenge in the present study (Fig. 6b, c, respectively).

Post-translational modification of intracellular proteins by O-linked N-acetylglucosamine (O-GlcNAc) in diabetes is a result of the excess glucose that drives the reaction. O-GlcNAc, in concert with ubiquitin, mediates several aspects of diabetic cardiomyopathy [49–53]. Therefore, we measured the amount of O-GlcNAc proteins in MuRF3−/− hearts, hypothesizing that the loss of MuRF3 cleared fewer O-GlcNAc-modified proteins to mediate the enhanced cardiomyopathy seen in vivo. Immunoblot analysis of O-GlcNAc-modified proteins in MuRF3−/− hearts demonstrated no differences from wildtype hearts when mice were fed a chow diet or 26 weeks of high fat diet (Additional file 2: Figure S2). While modest increases in O-GlcNAc levels were identified after 26 weeks of high fat diet, as expected with the observed hyperglycemia, differences in O-GlcNAc could did not appear to contribute to exaggerated MuRF3−/− cardiac dysfunction.

**Fig. 7:**
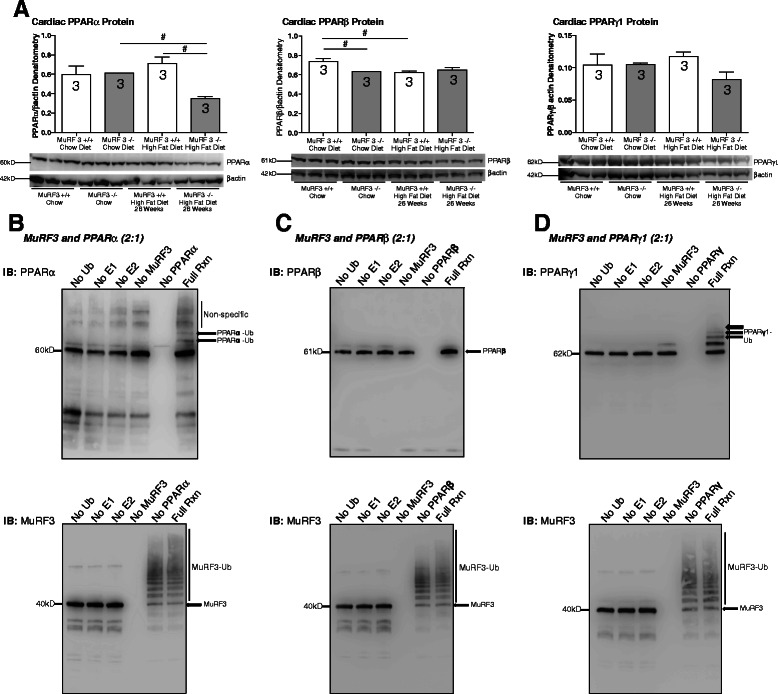
MuRF3 inversely regulates steady state levels of PPARα and PPARγ1. **a** Immunoblot analysis of cardiac LV PPARα, PPARβ, and PPARγ1 levels normalized to βactin. **b**-**d** In vitro ubiquitination assays of MuRF3’s ability to ubiquitinate PPARα (**b**), PPARβ (**c**), and PPARγ1 (**d**), with all lanes having Ub, E1, E2, MuRF3, and PPAR (=full reaction), unless otherwise indicated. Immunoblot for MuRF3 illustrates auto-ubiquitination (=MuRF3 activity) present in the same reaction as mono-ubiquitination (PPARα) and poly-ubiquitiation (PPARγ1). Values expressed as Mean ± SE of three independent experiments. A one-way ANOVA was performed to determine significance, followed by a Holm-Sidak pairwise comparison to determine significance between groups. N = 3/group. #p < 0.05

As a ubiquitin ligase, MuRF3 has been shown to ubiquitinate and degrade specific substrates in vivo and in vitro [10]. In mice with both MuRF1 and MuRF3 knocked out, accumulation of protein was identified histologically, later identified by mass spectrometry as beta/slow myosin [10]. Both MuRF1 and MuRF3 were then found to poly-ubiquitinate beta/slow myosin targeting it for degradation by the proteasome. One hypothesis we had in our current model of diabetic cardiomyopathy was that MuRF3 regulated cardiac PPAR activity by post-translationally modifying PPAR isoforms. To investigate this, we first determined the steady state levels of PPARα, PPARβ, and PPARγ1 in MuRF3−/− hearts by western blot analysis. Control MuRF3−/− hearts from mice on a chow diet did not have significantly different steady state protein levels of PPARα or PPARγ1 (Fig. 7a). Unexpectedly, PPARβ protein was significantly decreased in MuRF3−/− hearts. RT-qPCR analysis of PPARα, PPARβ, and PPARγ1 mRNA in MuRF3−/− hearts revealed that this baseline decrease in PPARβ could be explained by decreased PPARβ mRNA, suggesting that MuRF3 supported PPARβ through transcriptional regulation (Additional file 3: Figure S3A). After 26 weeks of high fat diet, MuRF3−/− hearts showed significantly decreased PPARα protein (Fig. 7a, left panel). However, no differences in PPARα mRNA were found (Additional file 3: Figure S3A). An alternative possibility is that compensatory expression of MuRF1 or MuRF2 may be occurring in the MuRF3−/− hearts, given their overlapping substrate specificities. However, hearts from control and 26 weeks high fat diet challenge do not have increased levels of MuRF1 or MuRF2 suggesting compensatory mechanisms (Additional file 3: Figure S3B). Contrary to our hypothesis that MuRF3 regulated PPAR activity through post-translational degradation, the expected accumulation of PPAR isoform(s) were not identified in MuRF3−/− hearts. Unexpectedly, in fact, decreases in PPARα and PPARβ were identified after high fat diet and at baseline, respectively (Fig. 7a).

We next investigated if MuRF3 may be regulating PPARα, PPARβ, and PPARγ1 through a non-degradatory mechanism through non-canonical (poly) ubiquitination mechanisms. To test MuRF3’s ability to ubiquitinate PPARs, recombinant ubiquitin, E1, E2 (UbcH5c), MuRF3 (E3), and PPARα were incubated and analyzed by western blot (Fig. 7b). Interestingly, a di-mono-ubiquitination was identified, with MuRF3’s autoubiquitination demonstrating the expected canonical poly-ubiquitination that leads to degradation in vivo. Similarly, MuRF3 di-mono-ubiquitinated PPARγ1 (Fig. 7d) but did not ubiquitinate PPARβ to any extent (Fig. 7c) with robust positive controls (MuRF3 autoubiquitination). Together, these studies suggest that MuRF3 has the ability to add two single ubiquitin moieties to PPARα and PPARγ1, but not PPARβ, which may regulate PPAR activity in vivo.

**Fig. 8:**
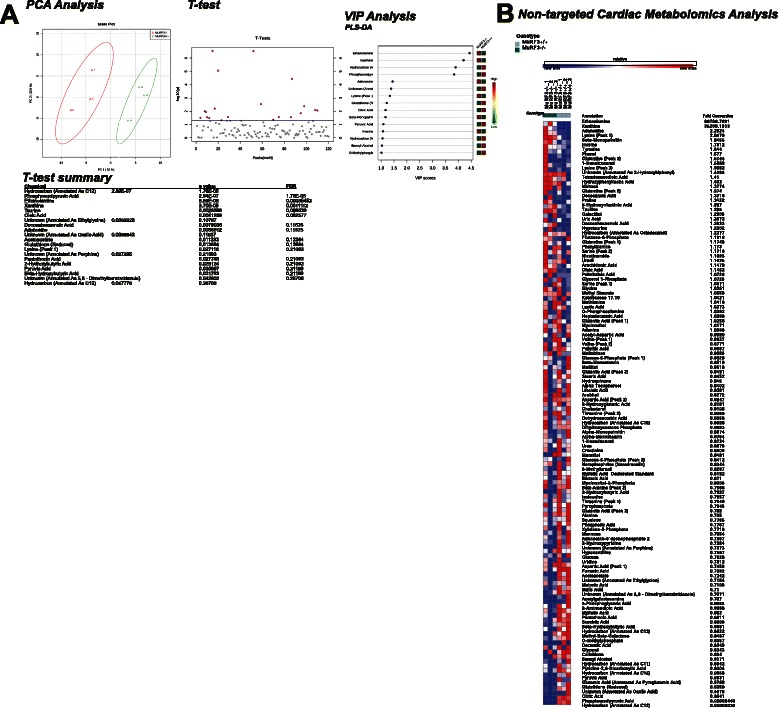
Non-targeted cardiac metabolomics of MuRF3−/− mice after 26 Weeks challenge with high fat diet. **a** Principal components analysis, *t*-test, and Variable Importance in the Projection Analysis/Partial Least Squares-Discriminant Analysis (PLS-DA), **b** Heat map of metabolites identified by non-targeted GC/MS analysis of cardiac tissue. N = 3/group. Significance determined as p < 0.05

By non-targeted metabolomics analysis, MuRF3−/− hearts after 26 weeks high fat diet clearly separated using Principal Components analysis (PCA) (Fig. 8a). Analysis of individual components (Fig. 8b) by *T*-test and Variable Interdependent Parameters (VIP) analysis identified specific metabolites that statistically were different from sibling wildtype control hearts (Fig. 8a, lower two panels). Further analysis of the *T*-test and VIP significant metabolites demonstrated enrichment for the peroxisome and mitochondria (Additional File 4: Figure 4Sa.), involving glutathione metabolism, taurine and hypotaurine metabolism, and the synthesis and degradation of ketone bodies (Additional File 4: Figure 4Sb.). The significant metabolites were enriched for pyruvate carboxylase deficiency, cardiogenic shock, non-insulin-dependent diabetes mellitus, and heart failure (Additional File 4: Figure 4Sc.).

## Discussion

We determined a role for MuRF3 in the development of diabetic cardiomyopathy characterized by the early development of heart failure and cardiac hypertrophy. Correlating with MuRF3−/− enhanced PPARα and PPARγ1 activities, MuRF3 ubiquitinated PPARα and PPARγ with di-mono-ubiquitin moieties. These non-canonical post-translational modifications do not result in substrate degradation, consistent with unaltered levels in the MuRF3−/− mice. Non-targeted metabolomics analysis further identified defects in MuRF3−/− hearts where altered metabolites were enriched similarly to those found in pyruvate decarboxylase deficiency, an enzyme with a PPAR-response controlling element [54].

The role of MuRF3 to date has focused on its structural role supporting the microtubule network [1], secondarily affecting its maintenance of sarcomeric Z and M-band formation due to these effects on tubulin dynamics [7]. MuRF3−/− mice challenged with myocardial infarction have shown a predisposition to cardiac rupture after MI [9]. Since microtubule stability is reported to contribute directly to the cardiac dysfunction observed in diabetic cardiomyopathy, loss of MuRF3 may contribute to the observed cardiac dysfunction in the current model [55–57]. Recent studies have implicated the activation of SRF in regulating growth during diabetic cardiomyopathy [58, 59]. Since MuRF3 interacts with SRF to inhibit its activity much in the same way its highly homologous MuRF1 and MuRF2 family members do [3, 5], we anticipated MuRF3−/− hearts would exhibit an exaggerated hypertrophy (HW/BW, LV Mass, etc.). To our surprise, MuRF3−/− hearts hypertrophied to the same degree as wildtype controls over time (Fig. 1d, Fig. 2e, Table 1), illustrating either MuRF3 minimal contribution to regulating SRF during the development of diabetic cardiomyopathy or potentially its redundant regulation of SRF with other MuRF family members.

This is the first published report of MuRF3’s upregulation during diabetic cardiomyopathy (Fig. 1c). Analysis of MuRF3’s promoter regulatory elements 10–20 kb upstream and 10 kb downstream have revealed multiple regions for the glucocorticoid receptor (GR), AML1, Lmo2, POU3F2, CREB, E2F, and E2F-1 (SAB Biosciences, http://www.genecards.org/cgi-bin/carddisp.pl?gene=TRIM54). Disease modeling and phenotypic drug screening for diabetic cardiomyopathy has revealed the potential for glucocorticoid receptor involvement [60], diabetic cardiomyopathy upregulation of CREB [61], and the E2F family transcription factors, including E2F1 [62]. Given the detrimental effects of inadequate MuRF3 during the development of type 2 diabetes seen in the MuRF3−/− mice, the upregulation of MuRF3 may be investigated further for its therapeutic potential suggested in the current studies.

The non-canonical ubiquitination with mono-ubiquitin moieties in the current study is distinctly different than previously reported canonical ubiquitination of PPARs in cancer cells (recently reviewed [63]). The canonical ubiquitination (non-Lys63 linked poly-ubiquitin chains) associated with mediating proteasome dependent degradation was not found in the present study. This non-degradatory ubiquitin mediated regulation of PPARs has not been reported in any system previously, including the heart. Similarly, specific ubiquitin ligases have not been identified in these processes prior to this identification of MuRF3. Contrasting to the current literature in cancer cells whereby poly-ubiquitination and/or degradation of substrate has been reported, the lack of degradation of PPARα and PPARγ1 despite mono-di-ubiquitination (Fig. 7b, 7D) differs significantly. The ubiquitin ligase 14ARF has been reported to di-ubiquitinate p53 in cancer cells in a manner which inhibits MDM2, another 14ARF substrate [64]. Like previous reports of multi-ubiquitinated (e.g., mono- and di-ubiquitination) substrates [65–67], MuRF3 does not lead to PPAR degradation in the physiological conditions. MuRF3’s multi-ubiquitination may offer additional clues into the complex regulation of cardiac PPAR isoforms previously unknown. Another novel finding in the present study is that cardiac MuRF3 supports the expression of PPARβ protein (Fig. 7a) at baseline through supporting its transcription (PPARβ mRNA, Additional file 3: Figure S3).

In our initial studies, we identified that nuclear PPARβ activity was increased 3 fold in MuRF3−/− hearts (Fig. 1a), but steady state protein levels were decreased (Fig. 7a) along with decreased PPARβ mRNA. Curiously, MuRF3 did not ubiquitinate PPARβ in vitro (Fig. 7c). Since the PPAR transcription factor involves a number of additional proteins, including RXRα and co-activators such as PGC-1, in addition to inhibitors, such as NCoR and SMRT, all of which can be SUMOylated and/or ubiquitinated [68], the answer may lie in the complexity and interactivity of the system. MuRF3−/− hearts may not be degrading a PPARβ enhancing co-factor, for example, to result in the enhanced PPARβ binding activity assayed in these studies. In this case, even the small decrease in PPARβ may not be enough to counteract the large increases in PPRE affinity this unknown factor may afford, giving us the results in the present study. Further molecular characterization of the PPARβ complex is warranted, given MuRF3’s interesting regulation of it both transcriptionally and through currently unknown activating factor(s).

Non-targeted metabolomics analysis of baseline MuRF3−/− hearts recently identified differences [14]. While few differences were seen in MuRF3−/− hearts compared to sibling wildtype controls, with significant overlap in PCA analysis, VIP significance identified taurine, α-monostearin, aldohexose1, and glutamic acid [14]. In contrast, we identified clear differences in the MuRF3−/− cardiac metabolomics signature compared to wildtype mice (Fig. 8a). While the taurine signature was again identified after high fat diet as it was on a chow diet, a broader array of metabolites were identified differentially in MuRF3−/− hearts (see VIP analysis metabolites and *T*-test summary, Fig. 8a).

In the diabetic heart, impaired glycolysis and facilitation of the pentose phosphate pathway has recently been described [69]. In a type 2 diabetes mellitus model (OLETF), pressure overloading has identified acute changes in inosine 5-monophosphate and adenosine, consistent with higher cardiac AMP deaminase activity and ATP depletion [69]. While such distinctive conclusions cannot be made based solely on the metabolomics findings, the altered inosine and adenosine has been identified in critically ICU patients [70] and may indicate underlying processes leading to the MuRF3−/− cardiac phenotype in the present study. Alternations in acetoacetate have been described in the heart, which has significance given the hearts ability to utilize acetoacetate by anaplerosis. The substitution of acetoacetate for glucose by anaplerosis, whereby TCA intermediates fill in, has been tested as an alternate pathway to form 2-oxoglutarate ex vivo [71]. This may be relevant to the MuRF3−/− phenotype, if applicable, as heart oxidizing acetoacetate resulted in decreased flux through 2-oxoglutarate dehydrogenase prior to contractile failure, therefore thought to be directly contributing to the changes in cardiac work [71].

## Conclusions

The present study suggests a protective role for MuRF3 in diabetic cardiomyopathy and an unexpected role in regulating fat storage despite being found only in striated muscle. MuRF3 is identified as an ubiquitin ligase that mono-ubiquitinates cardiac PPARα and PPARγ1 activities in vivo via post-translational modification, which appears to support PPAR stability in the context of diabetic cardiomyopathy.

## Additional files

Additional file 1: Figure S1. Analysis of circulating total cholesterol and triglyceride and histology in MuRF3−/− mice after high fat diet. **A.** Representative H&E analysis of MuRF2−/− and MuRF2+/+ tissue. **B.** Fasting total cholesterol and fasting serum triglyceride levels. **C.** Organ weights at 26 weeks high fat diet of gastrocnemius, soleus, and tibialis anterior. Values represent the mean ± SE (N indicated in bars). Values expressed as Mean ± SE. A one-way ANOVA was performed to determine significance followed by an all pairwise multiple comparison procedure (Holm-Sidak method). #p < 0.05.
Additional file 2: Figure S2. Detection of cardiac O-GlcNac Protein modifications in MuRF3−/− mice after 26 weeks HFD challenge. **A.** Densitometric analysis of O-GlcNac/βactin immunoblot (**B**). N = 3/group. Values expressed as Mean ± SE. A one-way ANOVA was performed to determine significance followed by an All Pairwise Multiple Comparison Procedure (Holm-Sidak method). #p < 0.05.
Additional file 3: Figure S3. mRNA analysis of cardiac PPAR isoform expression in MuRF3 −/− mice. Quantitative RT qPCR analysis of cardiac **A.** PPARα mRNA, PPARβ mRNA, and PPARγ1 mRNA and **B.** MuRF1 mRNA and MuRF2 mRNA at baseline and 26 weeks after high fat diet compared to sibling-matched wildtype hearts. N indicated in bars. A one-way ANOVA was performed to determine significance followed by an All Pairwise Multiple Comparison Procedure (Holm-Sidak method). #p < 0.05.
Additional file 4: Figure S4. Enrichment Analysis of Significantly different Metabolits Sets. **A.** Enrichment by Location-Associated Metabolite Sets **B.** Pathway Analysis of Metabolite Sets, and **C.** Disease-Associated Metabolite sets determined from VIP significant and t-test significant metabolites identified. N = 3/group.

## Abbreviations

EF%: Ejection fraction %
FS%: Fractional shortening %
LV: Left ventricular
LV Vol: Left ventricular volume
LVESD: Left ventricular end systolic dimension
LVEDD: Left ventricular end diastolic dimension
MuRF1/MuRF2/MuRF3: Muscle ring finger-1 (−2/-3)
O-GlcNAc: O-linked N-acetylglucosamine
PCA: Principal components analysis
PLS-DA: Partial least squares discriminant analysis
PPAR: Peroxisome proliferator activating receptor
SRF: Serum response factor
VIP: Variable interdependent parameters
