# Supplementary material for: Muscle ring finger-3 protects against diabetic cardiomyopathy induced by a high fat diet
Source: BMC Endocr Disord. 2015 Jul 28;15:36. doi: 10.1186/s12902-015-0028-z (PMC4515942; doi:10.1186/s12902-015-0028-z)
Supplement: Additional file 4: Figure S4. — Enrichment Analysis of Significantly different Metabolits Sets. A. Enrichment by Location-Associated Metabolite Sets B. Pathway Analysis of Metabolite Sets, and C. Disease-Associated Metabolite sets determined from VIP significant and t-test significant metabolites identified. N = 3/group. [file 12902_2015_28_MOESM4_ESM.pdf]

A Enrichment Analysis by Location

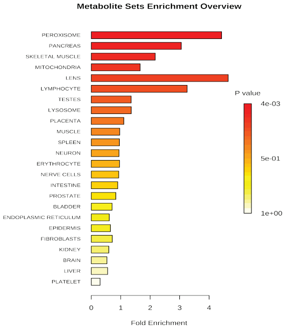

B Pathway Analysis

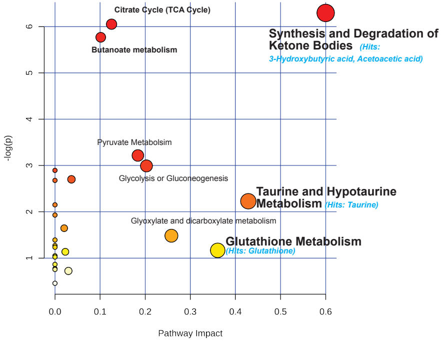

Metabolite Set	Total	Hits	Expected	p value	Holm P	Details
PEROXISOME	37	5	1.13	0.0039	0.222	Pyruvic acid, Sarcosine, Oxalic Acid, Taurine, Xanthine
PANCREAS	32	3	0.981	0.0699	1	Oxalic Acid, Inosine, Oleic Acid
SKELETAL MUSCLE	45	3	1.38	0.154	1	Xanthine, Oleic Acid, Sarcosine
MITOCHONDRIA	98	5	3.01	0.17	1	Glutathione, Adenosine, Pantothenic Acid, Pyruvic Acid, Sarcosine

Overview

C Enrichment by Disease-Associated Metabolite Sets

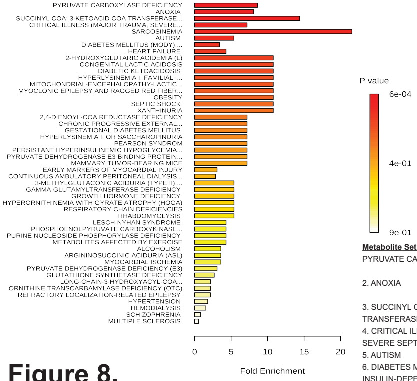

Metabolite Set	Total	Hits	Expected	p Value	Holm p	FDR	Details
PYRUVATE CARBOXYLASE DEFICIENCY	10	4	0.444	5.16E-4	0.177	0.177	*3-Hydroxybutyric acid, Acetoacetic acid, Pyruvic acid, Lysine*
2. ANOXIA	8	3	0.355	0.00368	1.0	0.63	*3-Hydroxybutyric acid, Acetoacetic acid, Pyruvic acid*
3. SUCCINYL COA: 3-KETOACID COA TRANSFERASE DEFICIENCY	3	2	0.133	0.00549	1.0	0.63	*3-Hydroxybutyric acid, Acetoacetic acid*
4. CRITICAL ILLNESS (MAJOR TRAUMA, SEVERE SEPTIC OR CARDIOGENIC SHOCK)	6	2	0.266	0.0253	1.0	1.0	*Adenosine, Inosine*
5. AUTISM	8	2	0.355	0.0447	1.0	1.0	*Adenosine, Glutathione*
6. DIABETES MELLITUS (MODY), NON-INSULIN-DEPENDENT	19	3	0.843	0.0463	1.0	1.0	*Citric acid, 3-Hydroxybutyric acid, Taurine*
8. HEART FAILURE	10	2	0.444	0.0681	1.0	1.0	*Taurine, 3-Hydroxybutyric acid*

Figure 8.
